# Supplementary material for: Global hypo-methylation in a proportion of glioblastoma enriched for an astrocytic signature is associated with increased invasion and altered immune landscape
Source: eLife. 2022 Nov 22;11:e77335. doi: 10.7554/eLife.77335 (PMC9681209; doi:10.7554/eLife.77335)
Supplement: Figure 2—source data 1. [file elife-77335-fig2-data1.zip › Figure_2_source_data_1/Figure_2I_J/homerResults/motif3.similar.html]

motif3

## Information for motif3

T
A
C
G
C
T
G
A
C
T
A
G
C
T
G
A
A
C
G
T
A
C
T
G
T
A
C
G
C
T
A
G
T
C
A
G
T
A
G
C
  
Reverse Opposite:  

A
C
T
G
A
G
T
C
A
G
T
C
A
G
T
C
A
G
T
C
G
T
C
A
A
G
C
T
A
G
T
C
G
A
C
T
A
T
G
C
  

|  |  |
| --- | --- |
| p-value: | 1e-52 |
| log p-value: | -1.208e+02 |
| Information Content per bp: | 1.805 |
| Number of Target Sequences with motif | 55.0 |
| Percentage of Target Sequences with motif | 7.58% |
| Number of Background Sequences with motif | 0.0 |
| Percentage of Background Sequences with motif | 0.00% |
| Average Position of motif in Targets | 89.0 +/- 55.1bp |
| Average Position of motif in Background | 0.0 +/- 0.0bp |
| Strand Bias (log2 ratio + to - strand density) | 0.0 |
| Multiplicity (# of sites on avg that occur together) | 1.02 |
| Motif File: | file (matrix) reverse opposite |

### Similar de novo motifs found

|  |  |  |  |  |  |  |  |
| --- | --- | --- | --- | --- | --- | --- | --- |
| Rank | Match Score | Redundant Motif | P-value | log P-value | % of Targets | % of Background | Motif file |
| 1 | 0.844 | C T A G C T A G C T G A T A C G A T G C A C G T A C T G C A T G T A C G T A C G T G A C G C T A | 1e-34 | -80.117812 | 7.30% | 0.95% | motif file (matrix) |
| 2 | 0.635 | A C G T C T A G T A C G C G T A C T A G T A C G C A T G T A C G T G A C A T G C | 1e-30 | -70.398536 | 5.23% | 0.67% | motif file (matrix) |
| 3 | 0.651 | A C G T A G T C A G T C A G T C C G T A A G T C A G T C A G T C A G T C A G T C | 1e-17 | -40.382795 | 4.82% | 0.95% | motif file (matrix) |
| 4 | 0.658 | A G T C A C T G A G T C A G T C A G T C A G T C A C T G A C G T A G T C A G T C A G T C A G T C | 1e-15 | -34.587662 | 4.41% | 1.06% | motif file (matrix) |
| 5 | 0.695 | A G T C A G T C A T G C A C G T A C G T A G T C A C G T A G T C | 1e-15 | -34.587662 | 4.41% | 1.01% | motif file (matrix) |
